# Epidermal growth factor receptor (EGFR) and Stat3 signal through Kras and have mutually opposite effects on Cten

**DOI:** 10.1186/1471-2164-15-S2-P59

**Published:** 2014-04-02

**Authors:** Saleh AlGhamdi, M ILyas

**Affiliations:** 1 King Abdullah International Medical Research Center (KAIMRC), Riyadh, KSA; 2 Division of Pathology, Nottingham University, Nottingham, UK

## Background

Cten is a protein located at focal adhesions and has been reported to be an oncogene in colon, breast, lung and gastric cancer [1-3]. We have previously shown than Cten is a target of K-ras in colorectal and pancreatic cancer [4]. In this study, we investigated whether two other proposed mechanisms i.e. EGFR and Stat3 signaling were involved in regulating Cten expression.

## Materials and methods

EGF was used to stimulate the EGFR while the PD153035 to inhibit the EGFR. On the other hand, IL-6 used to stimulate the STAT3 and siRNA for STAT3 was used for knockdown. The effect was confirmed using Western blot and QPCR and cell motility was assessed by transwell chambers.

## Results

Initially we manipulated EGFR signaling by (i) stimulation with EGF and (ii) inhibition by the PD153035 in the colorectal cancer cell lines SW620 and C32. In C32, EGF stimulation resulted in up-regulation of Kras and Cten whilst exposure to PD153035 resulted in down-regulation of both Kras and Cten. EGFR activation and inhibition was reflected by, respectively, increased and decreased cell motility although the effect of EGFR activation was lost by Cten Knockdown. In SW620, which harbours a KRAS mutation, modulating EGFR activity in this way had no effect on either Kras or Cten. Stat3 signaling has also been reported to positively regulate Cten. We tested this in SW620 by directly knocking down Stat3 and exposing cells to interleukin-6 (an activator of Stat3). Stat3 knockdown resulted in increased Cten whilst Stat3 activation resulted in downregulation of Cten. Testing for Kras expression showed that Stat3 was negatively regulating Kras and this was reflected in the Cten expression. Functional analysis however showed that inhibition of Stat3 resulted in a reduction of cell motility in a Kras and Cten-independent manner.

## Conclusions

We conclude that both EGFR signals through Kras to modulate Cten (and consequently ILK/FAK) and stimulates cell motility. Stat3 however negatively regulates Kras and consequently Cten although its net effect is to stimulate motility through an alternative mechanism.
